# Supplementary material for: Natural variation in reproductive timing and X-chromosome nondisjunction in Caenorhabditis elegans
Source: G3 (Bethesda). 2021 Sep 22;11(12):jkab327. doi: 10.1093/g3journal/jkab327 (PMC8664432; doi:10.1093/g3journal/jkab327)
Supplement: jkab327_Supplementary_Data [file jkab327_supplementary_data.zip › GENETICS-G3-2021-402755-s01.pdf]

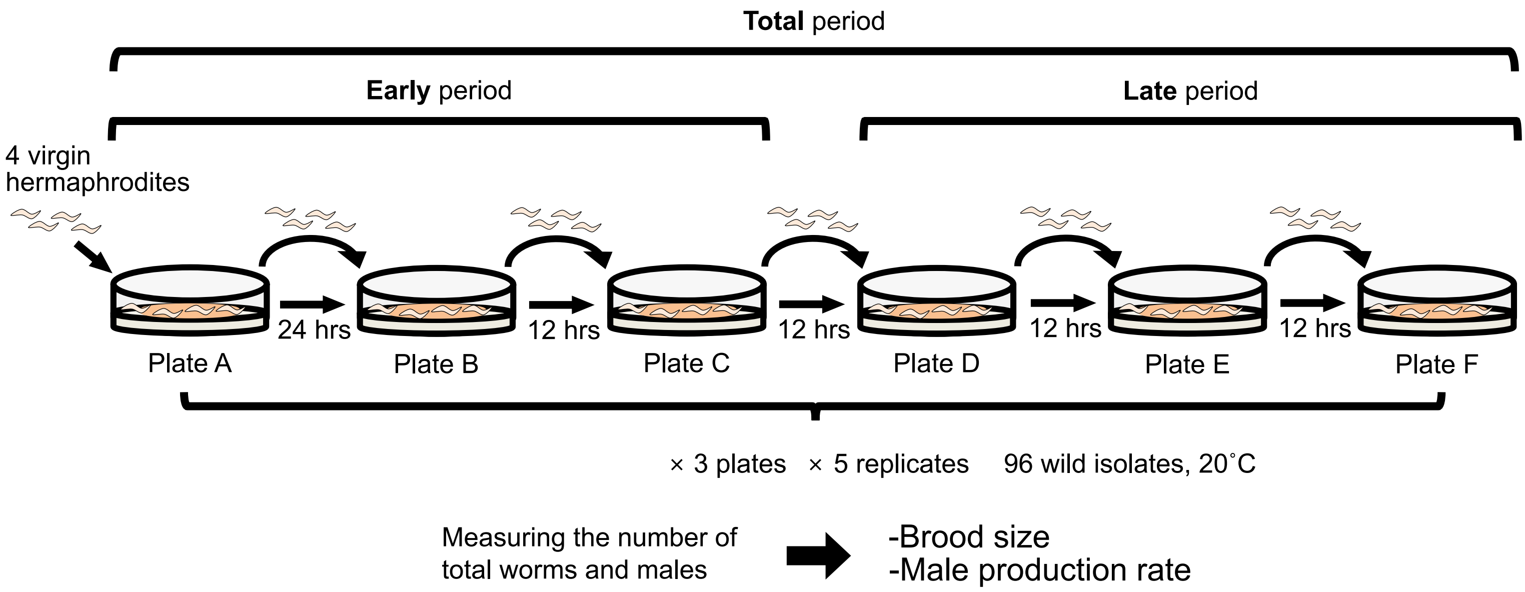

Total period
Early period
Late period
4 virgin
hermaphrodites
24 hrs
12 hrs
12 hrs
12 hrs
12 hrs
Plate A
Plate B
Plate C
Plate D
Plate E
Plate F
× 3 plates
× 5 replicates
96 wild isolates, 20˚C
Measuring the number of
total worms and males
-Brood size
-Male production rate
