# Supplementary material for: Natural variation in reproductive timing and X-chromosome nondisjunction in Caenorhabditis elegans
Source: G3 (Bethesda). 2021 Sep 22;11(12):jkab327. doi: 10.1093/g3journal/jkab327 (PMC8664432; doi:10.1093/g3journal/jkab327)
Supplement: jkab327_Supplementary_Data [file jkab327_supplementary_data.zip › GENETICS-G3-2021-402755-s04.pdf]

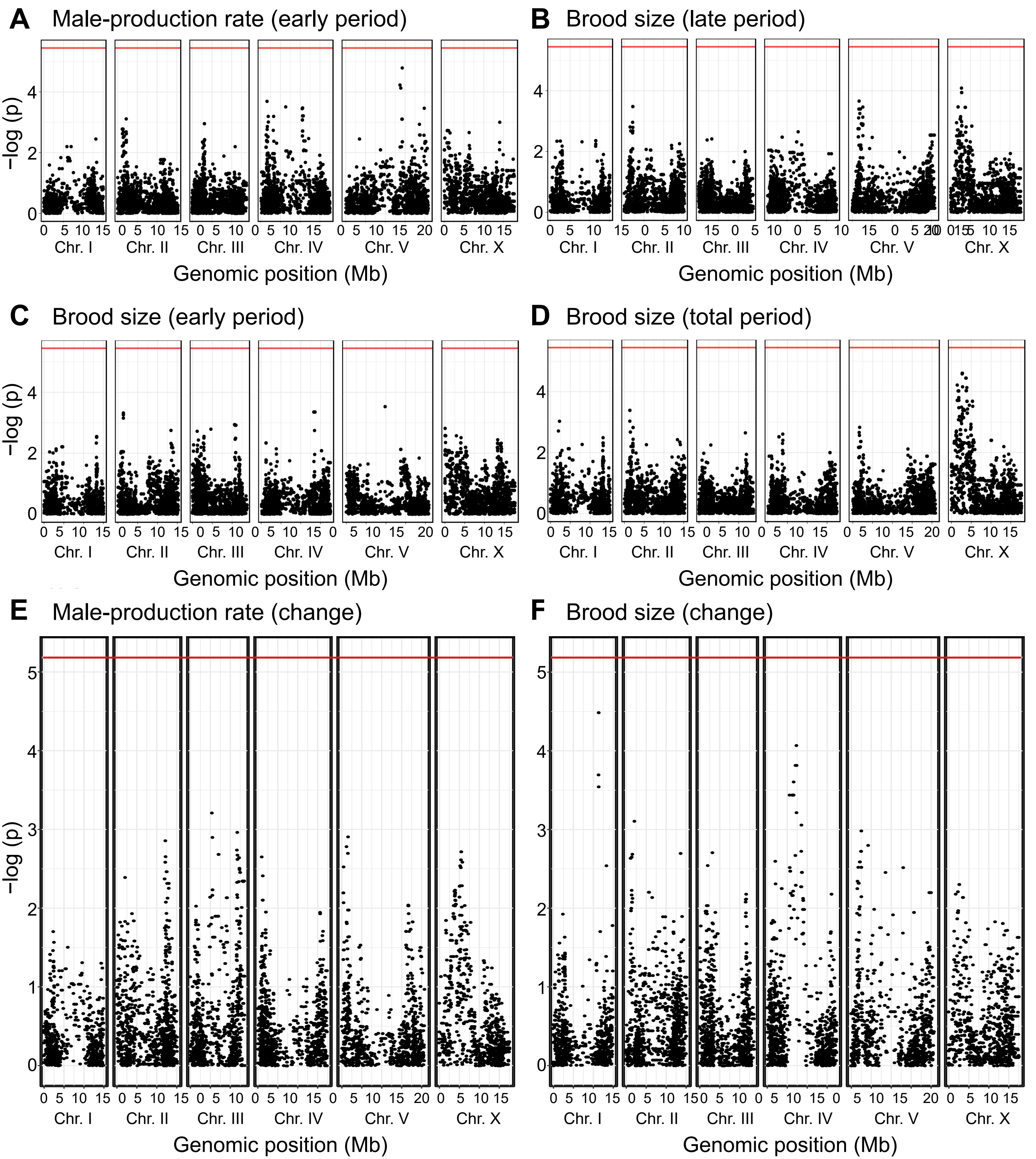

A Male-production rate (early period)
B Brood size (late period)
C Brood size (early period)
D Brood size (total period)
E Male-production rate (change)
F Brood size (change)
−log (p)
Chr. I
Chr. II
Chr. III
Chr. IV
Chr. V
Chr. X
Genomic position (Mb)
