# Supplementary material for: Natural variation in reproductive timing and X-chromosome nondisjunction in Caenorhabditis elegans
Source: G3 (Bethesda). 2021 Sep 22;11(12):jkab327. doi: 10.1093/g3journal/jkab327 (PMC8664432; doi:10.1093/g3journal/jkab327)
Supplement: jkab327_Supplementary_Data [file jkab327_supplementary_data.zip › GENETICS-G3-2021-402755-s10.docx]

**Supplementary Material**

**Figure S1.** Experimental scheme for measuring the brood size and male-production rate over time. The oocyte-production rates and X-nondisjunction rates were measured in two different reproductive periods. The earlier period was 48 hours after the last larval stage and the later period was the following 36 hours. Four unmated L4 hermaphrodites for each wild strain were raised on the first medium for 24 hours and transferred to fresh media every 12 hours for a total of 84 hours. As the last larval stage of C. *elegans* takes around 12 hours to develop into the adult stage, we used the first 48 hours to 36 hours after entering adulthood. We used three plates for each replicate and counted five replicates in total.

**Figure S2.** Phenotypic distributions of brood sizes and male-production rates, including outliers. Phenotypic variations for (A) brood size and (B) male-production rate in the total reproductive period including two outliers, ECA36 and NIC260 (arrowheads).

**Figure S3.** Block effects in brood size and male-production rate. The dots and scatter plots indicate phenotypic variations among blocks of CB4856, our internal control strain. Gray dots represent 15 replicates of each block, and blocks in total were compared. Blue dots indicate averages of replicates in each block; error bars, 95% confidence intervals. *P*-values were analyzed using one-way ANOVA.

**Figure S4.** Manhattan plots representing genome-wide association mapping results. (A) male-production rate of the early reproductive period. (B-D) Brood size of late (B), early (C), and total (D) reproductive periods. (E-F) Phenotypic changes between early and late reproductive periods of male-production rate (E) and brood size (F).

**Table S1.** Phenotypic values of brood size and male-production rate (%) over time for all wild isolates used in the project

**Table S2.** Independent samples t-test results between different experimental blocks of the CB4856 strain

**Table S3.** Pearson correlation coefficients between brood size and male-production rate of early, late and total reproductive periods and their changes over time.

**Table S4.** Candidate variants within genes which exhibit the Him phenotypes

**Table S5.** Candidate variants within genes which interact with the IIS receptor, DAF-2
