# Supplementary figures and images for: Natural variation in reproductive timing and X-chromosome nondisjunction in Caenorhabditis elegans
Source: G3 (Bethesda). 2021 Sep 22;11(12):jkab327. doi: 10.1093/g3journal/jkab327 (PMC8664432; doi:10.1093/g3journal/jkab327)

**A**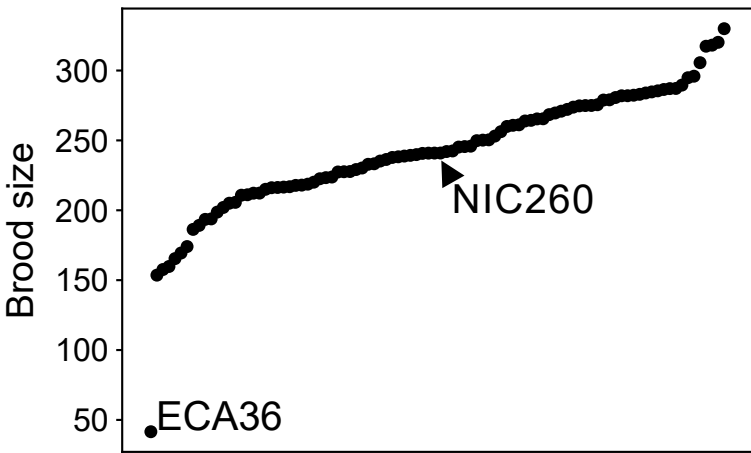**B**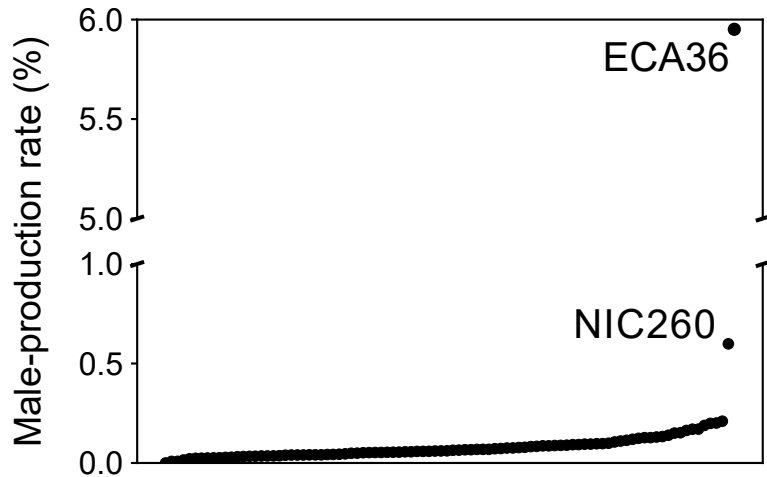

Supplement: jkab327_Supplementary_Data [file jkab327_supplementary_data.zip › GENETICS-G3-2021-402755-s02.pdf]

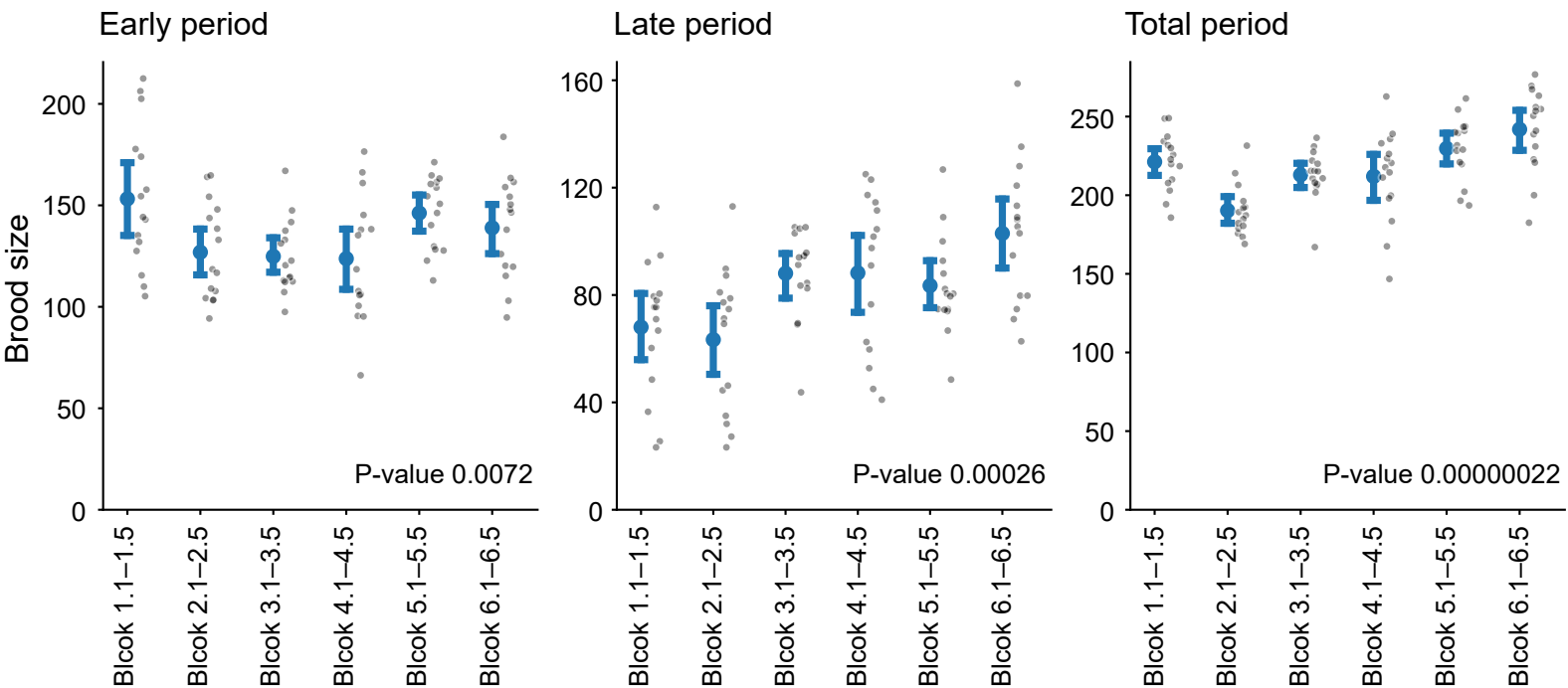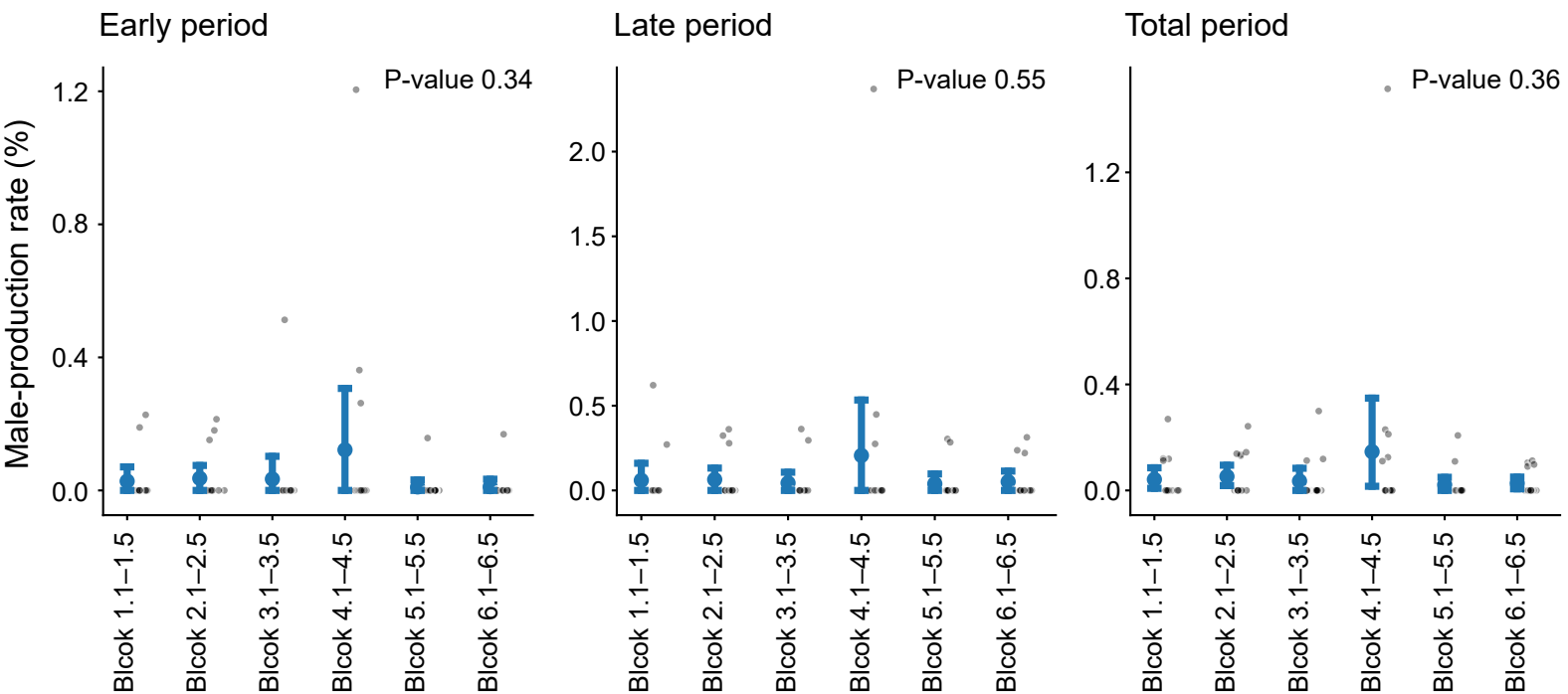

Supplement: jkab327_Supplementary_Data [file jkab327_supplementary_data.zip › GENETICS-G3-2021-402755-s03.pdf]
